# Time for a general approval of growth hormone treatment in adults with Prader–Willi syndrome

**DOI:** 10.1186/s13023-020-01651-x

**Published:** 2021-02-08

**Authors:** Charlotte Höybye, Anthony J. Holland, Daniel J. Driscoll, The Clinical and Scientific Advisory Board of The International Prader-Willi Syndrome Organisation

**Affiliations:** 1 Department of Endocrinology, Karolinska University Hospital and Department of Molecular Medicine and Surgery, Karolinska Institute, Stockholm, Sweden; 2 Department of Psychiatry, University of Cambridge, Cambridge, UK; 3 Division of Pediatric Genetics and Metabolism, Department of Pediatrics, University of Florida College of Medicine, Gainesville, FL USA

**Keywords:** Prader-willi syndrome, Growth hormone, Growth hormone treatment, Adults

## Abstract

Prader-Willi syndrome (PWS) is a complex, multi-system, neurodevelopmental disorder characterised by neonatal muscular hypotonia, short stature, high risk of obesity, hypogonadism, intellectual disabilities, distinct behavioural/psychiatric problems and abnormal body composition with increased body fat and a deficit of lean body mass. Growth hormone (GH) deficiency and other hormone deficiencies are common due to hypothalamic dysfunction. In children with PWS GH treatment has been widely demonstrated to improve body composition, normalise height and improve psychomotor development. In adults with PWS, GH’s main effects are to maintain normal body structure and metabolism. The positive effects of GH treatment on body composition, physical fitness and beneficial effects on cardiovascular risk markers, behaviour and quality of life in adults with PWS are also well established from several studies. GH treatment is approved for treatment of children with PWS in many countries, but until recently not as a treatment in young adults in the transition period or for adults in general. In this commentary we want to draw attention to the uneven global use of GH treatment, specifically in adults with PWS, and advocate for GH treatment to be approved internationally, not just for children, but also for adults with PWS and based only on the diagnosis of genetically confirmed PWS.

In the article “Three years of growth hormone treatment in young adults with Prader–Willi syndrome: sustained positive effects on body composition” Damen and colleagues [1] demonstrated that continued treatment with growth hormone (GH) in young adults with Prader–Willi syndrome (PWS) after attainment of final height maintained the improved body composition obtained with GH treatment during childhood. Following the proven success of GH treatment for children and some adults with PWS, it is time to expand upon the limited treatment options for adults with this condition and push for widespread approval of GH use across all ages.

PWS is a rare multi-system neurodevelopmental disorder caused by lack of expression of genes in the paternally inherited chromosomal region 15q11.2-q13 [2]. PWS is characterised by neonatal muscular hypotonia, short stature, a high risk for obesity, hypogonadism, intellectual disabilities, distinct behavioural/psychiatric problems and abnormal body composition with increased body fat and a deficit of lean body mass. GH deficiency (GHD) and other hormone deficiencies are common due to hypothalamic dysfunction [2, 3]. Furthermore, different nutritional phases have been described from feeding difficulties and failure to thrive in infancy, to progressive hyperphagia with high risk of severe obesity from childhood to adulthood [4]. Morbidity is increased and complications of obesity, such as respiratory and cardiovascular diseases, are major causes of death [2, 3].

The treatment options for adults with PWS are very limited and consist of a lifelong and environmentally controlled restricted energy intake, as well as regular physical activity and replacement of hormone deficiencies. For many years GH treatment in children with PWS has been approved in the USA, Europe and some other countries around the globe without the need for GH provocation testing, whereas in adults this approval has been obtained only recently and in only a few countries. In this commentary we want to draw attention to the uneven global distribution of approval for, and use of, GH as a treatment specifically for adults with PWS as reported to The International Prader–Willi Syndrome Organisation, and advocate for GH treatment to be approved internationally for adults with PWS and, for reasons we explain below, based only on the diagnosis of PWS.

In typically developing adults, GH no longer causes growth but it helps maintain normal body structure and metabolism, including keeping blood glucose levels within set levels. Treatment with GH in children with GHD started in the late 1950s. Assessment of efficacy consisted of measurement of height and body proportions, but in the late 1980s, when recombinant human GH became available, interest in the metabolic effects of GH intensified. The typical clinical picture of severe GHD in adults was described, and included abnormal body composition, fatigue, decreased physical activity, an adverse cardiovascular risk profile independent of obesity, and a reduced quality of life. Since the 1990′s adults, who have severe GHD according to specified diagnostic criteria, have been treated with GH and several studies have documented that GH improves body composition, cardiovascular risk factors and quality of life with few side effects [5].

Adults with PWS share similarities with non-PWS adults with GHD in particular regarding body composition given their reduced lean body mass and increased fat mass. Profound GHD, defined according to existing diagnostic criteria, has been demonstrated in up to 55% of adults with PWS [6, 7]. However, most GH stimulation tests measure the secretion of GH from the pituitary. This can lead to a false normal GH responses in patients whose GHD is of hypothalamic origin, such as in people with PWS. In addition, the increased amount of adipose tissue can confound the results. Levels of insulin growth factor I (IGF-I), the GH dependent growth factor, are low in the majority [6] and IGF-I levels are affected by nutritional status, presence of hypogonadism, spontaneous GH secretion and several other factors, which have not been fully investigated in PWS. Nevertheless, the hypothalamic dysfunction is permanent by nature of this condition, and therefore affected adults are expected to have the same hormone deficiencies as seen in the children. Thus, GHD is accepted as part of the phenotype PWS and due to all factors that might potentially affect the results of GH test in different directions, GH stimulation tests are of questionable value and are not of clinical relevance in this group of patients. Of note is the fact that GH stimulation tests might be cumbersome and difficult to perform in patients with PWS. Reasons for this include the presence of behavioural issues and the potential for the patient not cooperating. Furthermore, dependent on the amount of subcutaneous fat, blood sampling can be very difficult. Stimulation of GH secretion with insulin hypoglycemia is unpleasant and for an insulin resistant patient it might be difficult to obtain hypoglycemia.

GH treatment in children with PWS started in the 1990′s. Remarkable effects on height and body composition were observed and GH treatment in children with PWS was approved by the U.S. Food and Drug Administration and the European Medicines Agency independent of GH secretory status. The positive effects of GH treatment in adults with PWS are also well-known from several studies, as well as the recent article by Damen and colleagues [1, 6, 7]. In 2012 a meta-analysis of eight studies including 134 adults with PWS treated with GH showed that body composition improved with a weighted mean increase in LBM of 2.4 kg [95% CI 1.31;3.49] and a weighted mean reduction in body fat of − 2.9 kg [95% CI − 3.90; − 1.91] [8]. This positive effect on body composition was reported in all studies and has been confirmed in later studies, including long-term studies, all showing similar results [9–11]. However, BMI, waist circumference and waist-hip ratio were unchanged probably because the abnormal body composition in PWS is not accurately reflected by these measurements. The beneficial effect of GH on body composition has also been proven indirectly by the observation of impaired body composition after GH treatment was discontinued. Thus, 12 months of discontinuation of GH resulted in a 22% increase in body fat in young adults [12] and to an increase in visceral adipose tissue in adults from 25.2 ± 14.5 to 48.0 ± 26.1 cm^2^ (p = 0.024) [13]. In line with these findings, motor skills were impaired 6 months after GH treatment was discontinued [14]. Conversely, improvements in physical activity, as a result of the improved body composition resulting from GH treatment, have been observed. Using treadmill and accelerometers, exercise intensity was found to improve 16% after 6 months and 19% after 12 months of GH treatment in adults with PWS. [15]. Long-term GH treatment increased muscle strength by 13% and exercise endurance by 17% [16] and peak expiratory flow (PEF) by 12% [9]. Low bone mineral density and an increased frequency of fractures are frequently reported in adults with PWS. At present, it is not known for certain whether the low BMD is caused by impaired growth hormone secretion, low levels of sex-steroids, low muscular activity, low intake of calcium and D-vitamin or a combination of some or all of these things. Recently GH treatment in young adults with PWS did not improve BMD, probably because of a relatively short period of treatment and also the patients did not systematically receive sex hormone replacement [17]. On the other hand, in adults bone formation markers increased with GH treatment, whereas resorption markers did not change [18]. Moreover, it has been shown that GH treatment improved mental and cognitive function [14] and other studies have reported a better quality of life with GH treatment although caregivers/relatives were less optimistic in another study [19]. A significant impairment in psychosocial function in adults was noted when GH treatment was discontinued [14], while no change was seen in young adults [20]. Nevertheless, in PWS an integrated assessment of the patient is crucial in both considering a decision on the start of and in the evaluation of the effect of GH treatment, as also concluded in a large, recent study of 140 young adults with PWS [21].

In summary, the studies, including randomized controlled trials, have shown normalisation of IGF-I levels, increase in lean body mass, decrease in body fat, improved physical fitness, and beneficial effects on cardiovascular risk markers, and on behaviour and quality of life. These benefits have been observed in the absence of any relationship to levels of stimulated GH secretion. In addition, one study has clearly demonstrated that discontinuation of GH treatment at completion of growth in the transition period to adulthood, results in deterioration in body composition, and in endocrine and metabolic markers. These then normalize after resuming GH treatment [8].

While there are clear benefits one important caveat is the fact that GH is administered as daily subcutaneous injections. From our experience most adults do not manage the GH injections themselves, and supervision of the injection is needed. This implies a burden on the caregivers, but in the future this situation is likely to improve with the once weekly GH injections, which also have the potential to improve compliance [22]. Side effects to GH treatment in PWS were transient and included mild peripheral oedema, muscle pain and headache, similar to reports from other patient groups treated with GH. No worsening in scoliosis or sleep apnoea and no worsening or induction of diabetes mellitus were noted [7]. However, one of the physiological effects of GH is to increase glucose. An increase in glucose levels is therefore expected. For this reason, uncontrolled diabetes mellitus is a contraindication to GH treatment [6]. In the longer term the improvement in body composition achieved by GH treatment with the addition of the drugs being developed for appetite control might together lead to a substantial decrease in the risk of developing diabetes mellitus.

The incidence of psychoses in adults with PWS is high, particularly in those with the maternal uniparental disomy 15 (UPD) genetic type, and the presence of severe and active psychiatric illness is a contraindication to starting GH treatment at that point in time, but once the mental state is stabilized treatment with GH should be considered [6]. If the person is being treated with psychiatric medications the starting of GH may affect the metabolism of the medication and regularly psychiatric evaluation may be indicated for up to six months after GH is started.

PWS is a multi-system disorder and strict environmental control of food and regular physical activity remain the core interventions recommended for adults with PWS. The issue of GH treatment for adults is of significant concern to the PWS community. In fact, it was the single most-commonly asked question at the awareness booth hosted by the International Prader-Willi Syndrome Organisation (IPWSO) at the European Society of Paediatric Endocrinology meeting in 2019 and was also raised as a query at the European Congress of Endocrinology meeting in 2019. We argue that GH treatment, with its many beneficial effects and few side effects, offers an opportunity to relieve some of the adverse effects of PWS in adults. Given the reasons for GHD in people with PWS a different interpretation of test and treatment results from that in the typical population with GHD is in our opinion necessary. GH treatment is approved for treatment of children with PWS in many countries, but until recently not as a treatment in young adults in the transition period or for adults in general. The fact that GH treatment is now approved for adults with PWS in a few countries is therefore an important milestone. GH has an important role in optimisation of care in people with PWS, and we advocate for approval for continuing GH treatment in young adults with PWS based on the genetic diagnosis of PWS without testing of GH secretion. We also propose GH replacement should be offered to older adults with genetically confirmed PWS who did receive GH during childhood, but it was stopped when they reached adulthood, as well as those adults who never received GH during childhood. Hopefully we will see that soon.

## Data Availability

Not applicable.

## Abbreviations

GH: Growth hormone
GHD: Growth hormone deficiency
IGF-I: Insulin-like growth factor I
PWS: Prader-Willi syndrome
IPWSO: International Prader–Willi Syndrome Organisation
